# Zoledronic Acid Sensitizes Renal Cell Carcinoma Cells to Radiation by Downregulating STAT1

**DOI:** 10.1371/journal.pone.0064615

**Published:** 2013-05-31

**Authors:** Toshiki Kijima, Fumitaka Koga, Yasuhisa Fujii, Soichiro Yoshida, Manabu Tatokoro, Kazunori Kihara

**Affiliations:** Graduate School of the Department of Urology, Tokyo Medical and Dental University, Tokyo, Japan; Vanderbilt University, United States of America

## Abstract

Zoledronic acid (ZOL), a third-generation bisphosphonate that strongly inhibits osteoclast activity, is widely used for the treatment of bone metastasis from a variety of malignancies, including renal cell carcinoma (RCC). We previously reported that zoledronic acid (ZOL) clinically potentiates antitumor effects of radiotherapy (RT) on bone metastases from RCC. To date, however, it remains unknown whether ZOL radiosensitizes RCC and if it does, how. Here, we demonstrated that ZOL directly radiosensitizes RCC cells independent of osteoclast activity by potentiating the caspase-3-mediated apoptosis pathway. The radiosensitization by ZOL was observed in 786-O, A-498, and ACHN cells but not in Caki-1 cells. As its underlying molecular mechanism, we found that the signal transducer and activator of transcription 1 (STAT1) plays a key role. The three RCC cell lines, in which ZOL exerted a radiosensitizing effect, expressed STAT1 abundantly but Caki-1 cells did not. ZOL downregulated endogenous STAT1 expression in 786-O, A-498, and ACHN cells by a post-transcriptional modification. We confirmed that knockdown of endogenous STAT1 by siRNA sensitized 786-O cells to RT equivalently to ZOL, and that introduction of exogenous STAT1 rendered Caki-1 cells more RT-resistant. This is the first study to clarify the molecular mechanism by which ZOL directly radiosensitizes tumor cells. Because tumor cells commonly overexpress STAT1 and ZOL reportedly radiosensitizes various types of tumor cells, ZOL warrants further clinical and translational studies as a potent radiosensitizer against RT-resistant tumors overexpressing STAT1.

## Introduction

The standard of care for localized renal cell carcinoma (RCC) is surgical excision of the primary tumor. Recent studies have demonstrated that surgical resection of metastatic disease also contributes to improving the prognosis of patients with metastatic RCC [1]. Radiotherapy (RT) is also an indispensable therapeutic modality in controlling surgically unresectable metastases, particularly those to bone [2]. The major clinical problem with RT for RCC is resistance, which has been commonly recognized by clinicians [3]. Although previous basic research has demonstrated the potential molecular mechanisms underlying the RT resistance of RCC [4]–[6], the findings have not led to any significant improvement in therapeutic strategies in clinical practice. Thus, clinically oriented translational research on the mechanisms of RT resistance is essential to the development of a novel strategy that improves RCC response to RT.

Bone is one of the most frequent metastatic sites from RCC, accounting for approximately 30% of all metastatic sites [7]. These bone lesions are predominantly osteolytic and cause considerable skeletal-related events (SREs), including pathologic fracture and spinal cord compression, which significantly impair patient quality of life [7]. RT to bone metastasis often relieves pain but rarely results in radiological objective response or reduced risk of SREs [8]. Zoledronic acid [ZOL; 2-(imidazol-1-yl)-hydroxy-ethylidene-1,1-bisphosphonic acid], a third-generation amino-bisphosphonate, is a potent inhibitor of osteoclast activity that has been widely used for the management of bone metastases from various malignancies, including RCC [9]. Although ZOL as a single agent reportedly decreased the risk of SREs and prolonged the SRE-free survival in RCC patients with bone metastases, the objective response rate was quite low (7%) and more than half of the patients eventually experienced SREs [10]. Recently, we and another group reported that ZOL potentiates RT effects on bone metastases from RCC [11], [12]. In our study, the combination therapy yielded a significantly higher objective response rate (60%) and longer median SRE-free survival (median not reached) compared to RT alone (8% and 18.7 months, respectively) [11].

In addition to the inhibition of osteoclast activity, ZOL has been demonstrated to exert direct antitumor effects on various tumors, including RCC [13]. Thus, ZOL may directly radiosensitize RCC cells at bone metastasis sites.

In the current study, we demonstrated that ZOL directly sensitizes RCC cells to RT independent of osteoclast activity. As its underlying molecular mechanism, ZOL post-transcriptionally downregulates the signal transducer and activator of transcription 1 (STAT1), which is responsible for the radiosensitization of RCC cells.

## Materials and Methods

### Reagents, Antibodies, and Cell Lines

ZOL was obtained from Novartis Pharma AG (Basel, Switzerland). Primary antibody against STAT1, phospho-STAT1 (Tyr701), Erk, phospho-Erk (Thr202/Tyr204), Akt, phospho-Akt (Ser473), caspase-3, cleaved caspase-3, Ras, β-actin (Cell Signaling Technology, Danvers, MA, USA), and the unprenylated form of Rap1A (Santa Cruz Biotechnology, Santacruz, CA, USA) were used for western blot analyses. Four human RCC cell lines, 786-O (CRL-1932), Caki-1 (HTB-46), A-498 (HTB-44), and ACHN (CRL-1611), were obtained from the American Type Culture Collection and cultivated in RPMI 1640 supplemented with 10% heat-inactivated fetal bovine serum and 50 U/ml of penicillin and 50 µg/ml streptomycin at 37°C and 5% CO_2_. The cells were plated and cultured to achieve 80% confluence on the day of experiments.

### Cell Proliferation Assay

To assess the anti-proliferative effect of ZOL on RCC cell line, 3-(4,5-dimethylthiazol-2-yl)-5-(3-carboxymethoxyphenyl)-2-(4-sulfophenyl)-2H-tetrazolium (MTS) assay was performed. RCC cell lines were seeded in triplicate at 2×10^3^ cells in 100 µl of medium per well in 96-well plates, and allowed to grow for 24 h. Then, the cells were treated with various concentrations of ZOL (range, 0 to 50 µM) for 24, 48, and 72 h. Thereafter, 10 µl of the Cell Titer 96 Aqueous One Solution (Promega, Madison, WI, USA) was added to each well. After incubation with the reagent for 1 h, the absorbance at 490 nm was measured using a Sunrise microplate reader (TECAN, Mannedorf, Switzerland).

### Western Blot Analysis

Total proteins were extracted using TNESV lysis buffer (50 mM Tris-HCl (pH 7.4), 1% Nonidet P-40, 1 mM EDTA, 100 mM NaCl, 1 mM Na_3_VO_4_) supplemented with complete mini protease inhibitors (Roche Applied Science, Mannheim, Germany). Equal amounts of protein were separated on 5 to 15% (for STAT1, Erk and Akt) or 15% (for caspase-3, Ras and Rap1A) SDS polyacrylamide gels, transferred to nitrocellulose membranes, and probed with antibodies. Protein expression was visualized with the enhanced chemiluminescence detection system (Pierce, Rockford, IL, USA) using an LAS-4000 imager (Fujifilm, Tokyo, Japan) and densitometrically quantified with Multi Gauge v3.2 software (Fujifilm).

### Radiation

Cells were exposed to ionizing radiation using an industrial X-ray generator, HS-225 (Shimadzu, Tokyo, Japan) operated at 225 V, 13 mA with 1-mm Cu filtration to achieve a dose rate of 0.83 Gy/min. Cells of a non-irradiated control group were mock treated in the same environment.

### Clonogenic Cell Survival Assay

Cells irradiated at a dose of 0, 4, and 8 Gy were immediately seeded into 100-mm tissue culture dishes at multiple cell concentrations. Fourteen days after seeding, colonies were stained with Giemsa, and the number of colonies consisting of at least 50 cells was counted. Plating efficiency (number of colonies counted/number of seeded cells) and survival fraction (the ratio of plating efficiency of each treatment to that of non-irradiated control) were calculated. Individual assays were repeated twice.

### Transfection

To knockdown endogenous STAT1 expression, STAT1-specific siRNA (Cell Signaling Technology) was introduced into 786-O cells expressing STAT1 abundantly. Under 80% confluence, STAT1-specific or control siRNA was transfected using HiPerFect Transfection Reagent (QIAGEN, Hilden, Germany) according to the manufacturer’s instruction. To evaluate the contribution of STAT1 overexpression to RT resistance, Caki-1 cells expressing STAT1 faintly were transiently transfected with the pBR322-based vector carrying full-length protein coding sequence of STAT1 (eGFP STAT1 WT, Addgene, Cambridge, MA, USA) or the GFP coding region as a control (pAcGFP-C1, Clontech, Mountain View, CA, USA). The transfection into Caki-1 cells was performed under 80% confluence using FuGENE HD transfection Reagent (Roche Applied Science, Mannheim, Germany) according to the manufacturer’s instruction. RT was given 48 h after transfection.

### Reverse-transcriptional PCR and Quantitative Real-time PCR

786-O cells were exposed to ZOL at 10 µM for 24 and 48 h and total RNA was extracted using NucleoSpin RNA II kit (Macherey-Nagel, Düren, Germany) following the manufacturer’s instruction. Total RNA of 2 µg was reverse-transcribed into first-strand cDNA using the ThermoScript RT system (Invitrogen, Carlsbad, CA, USA) at the final volume of 20 µl. Quantitative real-time PCR was performed using the LightCycler system and the SYBR green I dye (Roche Applied Science). The primers of STAT1 and glyceraldehydes-3-phosphate dehydrogenase (GAPDH) were purchased from Search-LC (Heidelberg, Germany). The PCR conditions were as follows: initial denaturation at 95°C for 10 min, followed by 35 cycles of denaturation at 95°C for 10 s, annealing at 68°C for 10 s, and extension at 72°C for 16 s. Fluorescent product was measured by a single acquisition mode after each cycle.

## Results

### Anti-proliferative Effects of ZOL on RCC Cell Lines

**Figure 1:**
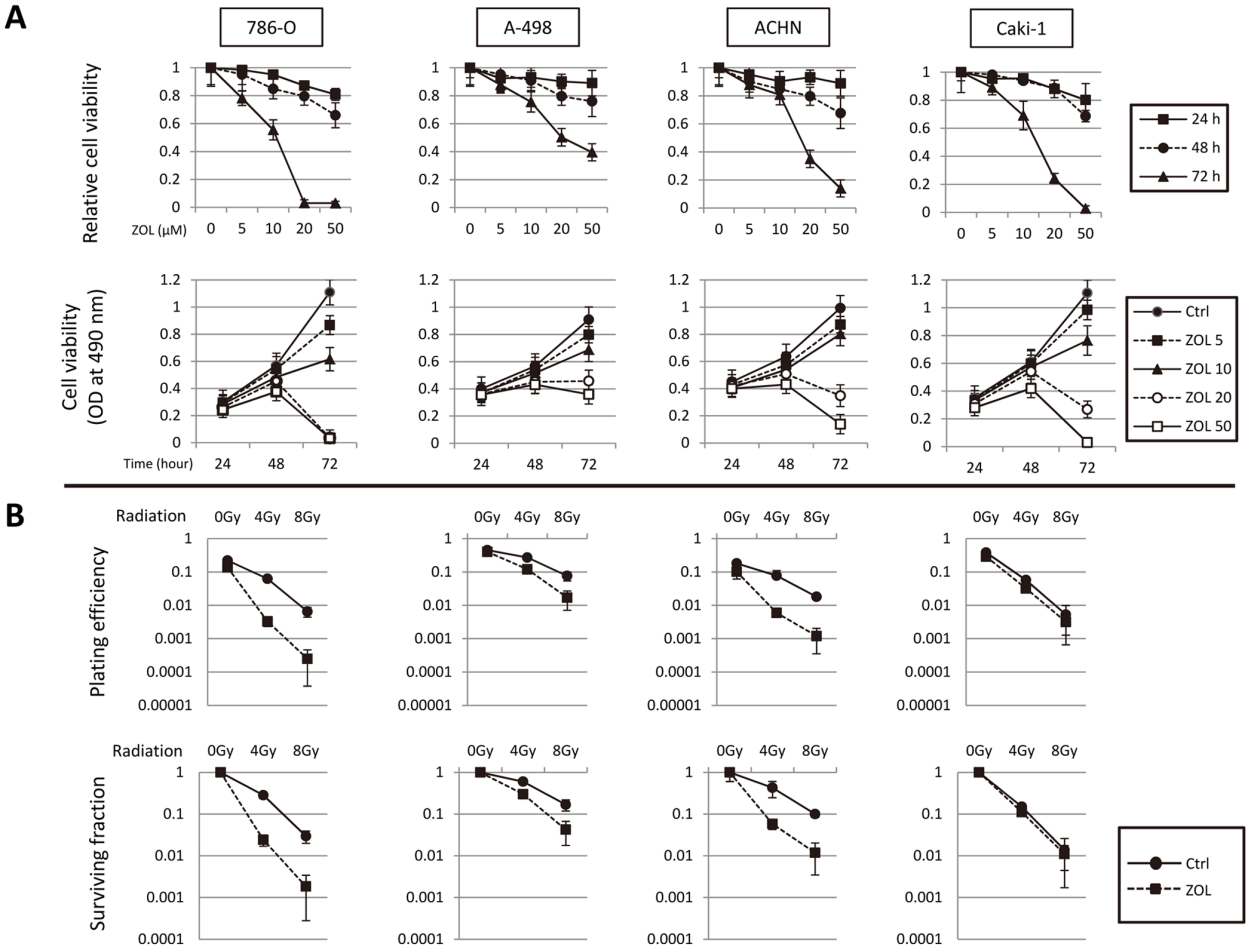
ZOL directly exerts antiproliferative and radiosensitizing effects on RCC cells. *A,* antiproliferative activity of ZOL against RCC cells. Dose-dependent curves of relative cell viability according to various exposure times (upper panels) and time-course curves of absolute cell viability according to various ZOL concentrations (lower panels) were evaluated for four RCC cell lines (786-O, A-498, ACHN, and Caki-1 cells). Cells treated with ZOL at indicated concentrations for indicated durations were evaluated for cell viability using MTS assay. Data represent mean ± standard deviation (SD) from three independent experiments. *B,* radiosensitizing effect of ZOL on RCC cells. Cells pretreated with ZOL at 10 µM (786-O and Caki-1) or 20 µM (A-498 and ACHN) for 48 h were irradiated. Fourteen days after plating the cells, plating efficiency (upper panels) and survival fraction (lower panels) were calculated by means of clonogenic cell survival assay. Data represent mean ± SD from two independent experiments.

Growth inhibitory effects of ZOL were examined using MTS assay. ZOL inhibited growth of four RCC cell lines (786-O, A-498, ACHN, and Caki-1) examined in a time- and dose-dependent manner (Fig. 1A, upper panels). Exposure to ZOL for 72 h exhibited a cytostatic effect at lower concentrations (10 µM or less) but a cytocidal effect at higher concentrations (20 µM or greater) on all four cell lines (Fig. 1A, lower panels).

### ZOL Directly Radiosensitizes RCC Cells Independent of Osteoclast Activity

We investigated whether ZOL directly radiosensitizes RCC cells in the absence of osteoclasts. The four RCC cell lines were pretreated with or without ZOL at 10 µM (786-O and Caki-1) or 20 µM (A-498 and ACHN) for 48 h; treatment with ZOL at these concentrations for 48 h does not exhibit a cytocidal effect based on the results of MTS assay (Fig. 1A, lower panels). Cells were then irradiated at indicated doses in Fig. 1B. ZOL alone did not decrease the plating efficiency of non-irradiated RCC cell lines. ZOL significantly potentiated RT effects on 786-O, A-498, and ACHN cells but not on Caki-1 cells. Thus, ZOL directly radiosensitizes RCC cells and exertion of the radiosensitizing effect appears to depend on cell lines.

### ZOL Potentiates RT-induced Activation of Caspase-3

**Figure 2:**
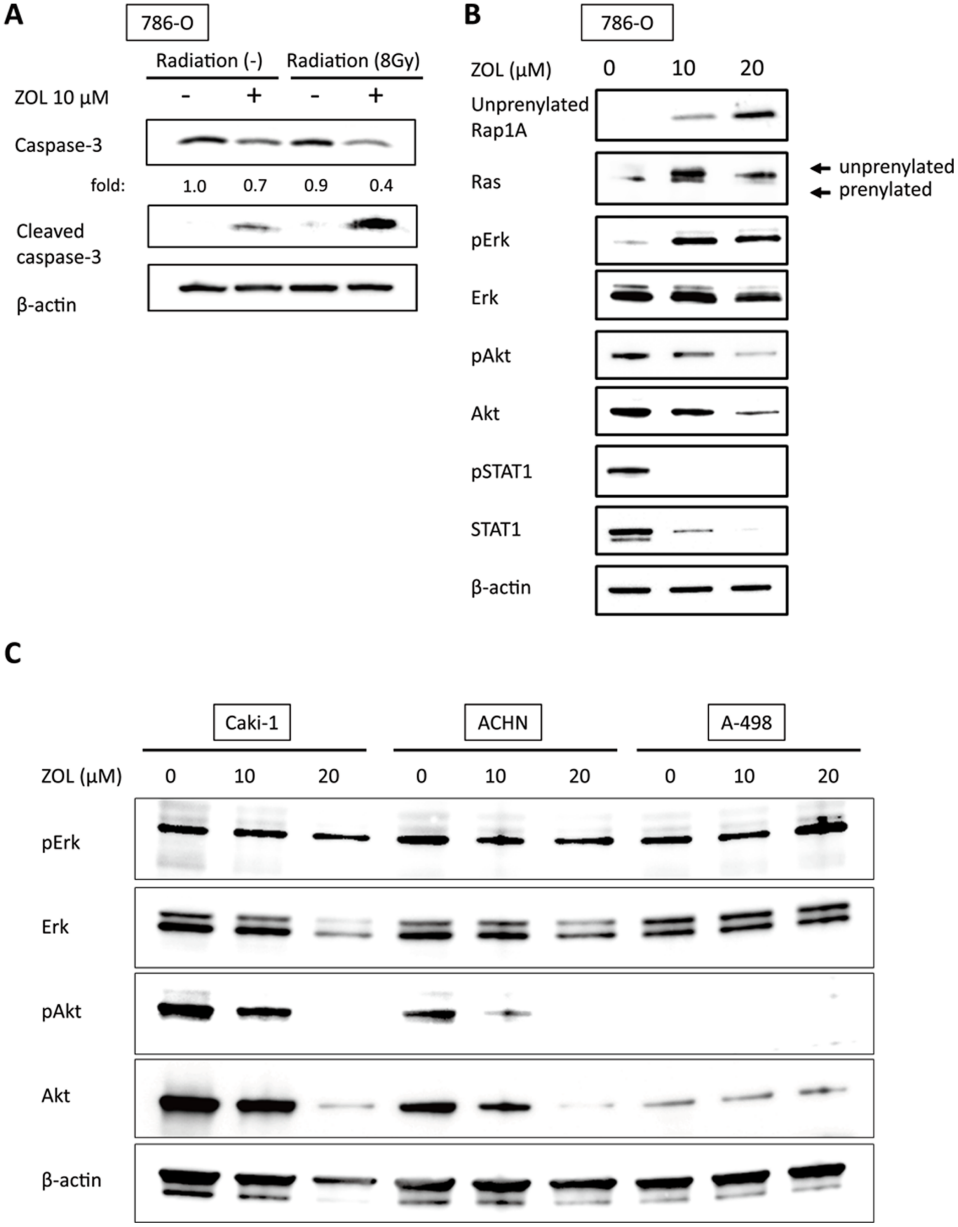
ZOL potentiates RT-induced caspase-3 activation and modulates Ras-mediated signalings and STAT1 expression in RCC cells. *A,* ZOL potentiates RT-induced caspase-3 activation in 786-O cells. Cells pretreated with or without ZOL at 10 µM for 48 h underwent RT at 8 Gy and cell lysates were subjected to western blot 48 h after RT. *B,* effect of ZOL on the STAT1 expression and other signal transduction pathways in 786-O cells. After 48 h exposure to ZOL (0, 10, and 20 µM), cell lysates were collected for western blot. ZOL significantly decreased STAT1 levels in a dose-dependent manner. ZOL inhibited prenylation of Ras and Rap1A in a dose-dependent manner. In the downstream of Ras, Erk phosphorylation increased whereas Akt activity decreased mainly due to the decrease in total Akt level. *C,* effects of ZOL on Erk and Akt in Caki-1, ACHN, and A-498 cells. Phospho-Erk level was almost stable in Caki-1 and ACHN and was slightly increased in A-498 cells after exposure to ZOL. Similar to 786-O cells, ZOL downregulated phospho- and total Akt in Caki-1 and ACHN but not in A-498 cells with scant basal Akt activity.

To clarify whether the radiosensitization by ZOL is associated with induction of apoptosis, we examined the effect of ZOL alone, RT alone, and their combination on caspase-3 activity. 786-O cells pretreated with or without 10 µM ZOL for 48 h underwent RT at 8 Gy. Caspase-3 activity was measured 24 h after RT. As shown in Fig. 2A, ZOL alone and RT alone slightly activated caspase-3. Their combination exerted greater caspase-3 activation than ZOL alone or RT alone, suggesting that ZOL enhances RT-induced apoptosis by activating caspase-3 in RCC cells.

### Effects of ZOL on Ras-mediated Signaling Pathways and STAT1

To clarify molecular mechanisms by which ZOL radiosensitizes RCC cells, we first evaluated the effects of ZOL on Ras-mediated signaling pathways in 786-O cells. ZOL inhibits prenylation of Ras [14] and accordingly Ras-mediated signaling pathways, including Erk [15] and PI3K/Akt [16] pathways, which are potentially associated with intrinsic RT resistance of tumor cells [17]. In agreement with previous reports [13], [18], ZOL inhibited prenylation of small G-proteins, Ras and Rap1A, in a dose-dependent manner (Fig. 2B). Despite unprenylation of Ras in the upstream, ZOL activated Erk in a dose-dependent manner. ZOL attenuated Akt activity in a dose-dependent manner, mainly by downregulating the total Akt level. Because inhibition of the Ras pathway by ZOL could not simply explain the Erk activation and total Akt downregulation observed in 786-O cells, the effects of ZOL on Erk and Akt were examined in Caki-1, ACHN, and A-498 cells (Fig. 2C). Phospho-Erk level was almost stable in Caki-1 and ACHN and was slightly increased in A-498 cells. Both total and phosphorylated Akt levels were reduced in Caki-1 and ACHN but not in A-498 cells with scant basal Akt activity.

STAT1 overexpression was recently reported to be associated with intrinsic RT resistance of tumors [19]. We evaluated the effects of ZOL on STAT1 expression in 786-O cells (Fig. 2B). 786-O cells expressed STAT1 and its phosphorylated form at the basal level. Intriguingly, ZOL decreased STAT1 remarkably in a dose-dependent manner; it also decreased its phosphorylated form. This prompted us to investigate the role of STAT1 in ZOL-mediated radiosensitization of RCC cells.

### ZOL Downregulates STAT1 Expression Post-transcriptionally

**Figure 3:**
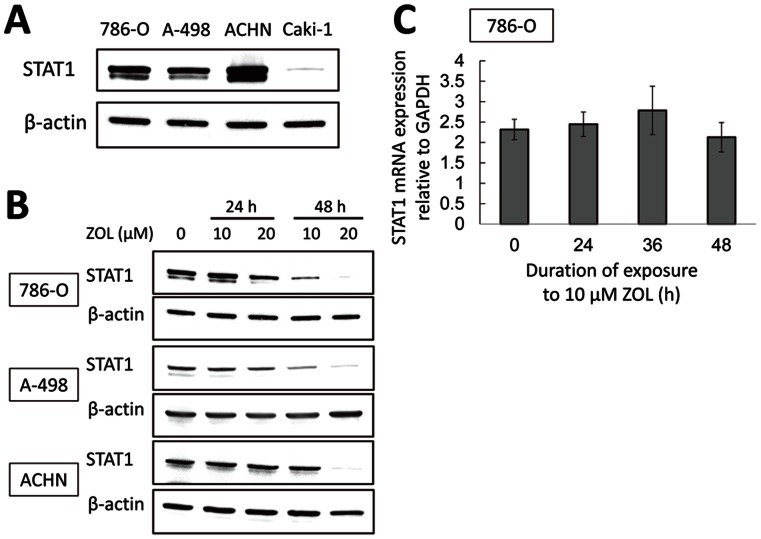
ZOL downregulates STAT1 expression posttranscriptionally. *A,* baseline expression of STAT1 in RCC cell lines. Cell lysates from four untreated RCC cell lines (786-O, A-498, ACHN, and Caki-1) were subjected to western blot for STAT1 expression. *B,* ZOL attenuates STAT1 expression at protein level in RCC cells expressing STAT1 (786-O, A-498, and ACHN). Cells treated with ZOL at indicated concentrations for 24 and 48 h were subjected to western blot for STAT1 expression. *C,* ZOL does not downregulate STAT1 expression at mRNA level. 786-O cells treated with ZOL at 10 µM for indicated durations were subjected to quantitative real-time RT-PCR. Data represent mean ± SD from two independent experiments.

The radiosensitizing effect of ZOL was observed in 786-O, A-498, and ACHN cells but not in Caki-1 cells. To investigate the association between the effect of ZOL and STAT1 expression, the baseline expression of STAT1 was compared between the four RCC cell lines. Notably, 786-O, A-498, and ACHN exhibited high baseline expression of STAT1, whereas Caki-1 expressed it only faintly (Fig. 3A). ZOL decreased STAT1 expression in a time- and dose-dependent manner in the three cell lines expressing STAT1 abundantly (Fig. 3B). Next, we examined whether ZOL downregulates STAT1 at the transcriptional level. Treatment with ZOL at 10 µM up to 48 h, which remarkably attenuates STAT1 expression at protein level in 786-O cells, resulted in no significant change in STAT1 mRNA levels (Fig. 3C). This indicates that ZOL downregulates STAT1 by a post-transcriptional modification.

### Role of STAT1 in RT Sensitivity of RCC Cells

**Figure 4:**
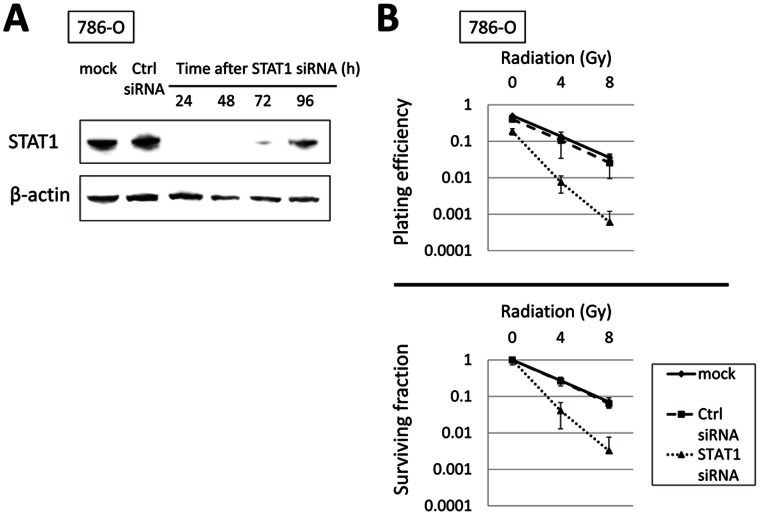
Knockdown of endogeneous STAT1 by siRNA radiosensitizes RCC cells equivalently to ZOL. *A,* time course of endogeneous STAT1 expression after STAT1-specific siRNA transfection in 786-O cells. STAT1 siRNA disrupted expression of >90% of endogenous STAT1 protein 24 to 72 h after transfection. *B,* knockdown of endogeneous STAT1 radiosensitizes 786-O cells equivalently to ZOL. Cells transfected with control or STAT1 siRNA were irradiated at indicated doses 48 h after transfection. Fourteen days after plating the cells, plating efficiency (upper panel) and survival fraction (lower panel) were calculated by means of clonogenic cell survival assay. Data represent mean ± SD from two independent experiments.

To investigate whether STAT1 plays a critical role in RT sensitivity of RCC cells, we silenced the endogenous STAT1 expression in 786-O cells and evaluated their RT sensitivity. As shown in Fig. 4A, transfection of STAT1 siRNA disrupted the expression of >90% of endogenous STAT1 protein 24 to 72 h after transfection. Clonogenic assay was performed for the cells irradiated 48 h after transfection with STAT1 or control siRNA. As depicted in Fig. 4B, STAT1 knockdown radiosensitized 786-O cells equivalently to treatment with ZOL at 10 µM for 48 h (cf. Fig. 1B). Thus, loss of endogenous STAT1 expression increases RT sensitivity in RCC cells.

**Figure 5:**
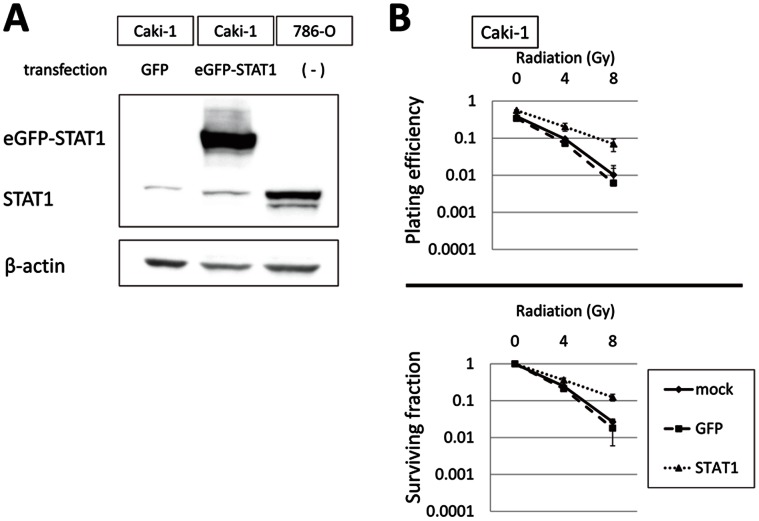
Introduction of exogeneous STAT1 increases RT resistance in RCC cells lacking endogeneous STAT1 expression. *A,* Exogenous STAT1 expression after transfection with a vector carrying GFP or eGFP-fused full-length STAT1 cDNA in Caki-1 cells. Cells efficiently expressed exogenous STAT1 at 48 h after transfection. *B,* introduction of exogenous STAT1 increases RT resistance in Caki-1 cells. Cells transfected with vectors carrying GFP or eGFP-STAT1 cDNA were irradiated at indicated doses 48 h after transfection. Fourteen days after plating the cells, plating efficiency (upper panel) and survival fraction (lower panel) were calculated by means of clonogenic cell survival assay. Data represent mean ± SD from two independent experiments.

Lastly, we examined whether the exogenous introduction of STAT1 increases RT resistance in Caki-1 cells lacking STAT1 expression. As depicted in Fig. 5A, introduction of eGFP-STAT1 cDNA increased exogenous STAT1 level at 48 h after transfection. Caki-1 cells were incubated for 48 h after introduction of eGFP-STAT1 or GFP cDNA, irradiated, and subjected to clonogenic assay. As expected, exogenous expression of STAT1 increased the RT resistance of Caki-1 cells (Fig. 5B). These results reveal that STAT1 plays a key role in regulating the RT sensitivity of RCC cells.

## Discussion

Based on the clinical efficacy of the combination therapy with ZOL and RT against bone metastases from RCC, we hypothesized that ZOL sensitizes RCC to RT directly and/or through modifying osteoclast activity. The current study clearly demonstrated that 10 µM of ZOL exerts radiosensitizing effects directly on RCC cells by enhancing RT-induced caspase-3 activation. As an underlying molecular mechanism, we found that ZOL attenuates endogenous STAT1 expression in RCC cells by a post-transcriptional modification. We further confirmed that STAT1 plays a key role in the RT sensitivity of RCC cells. This is the first study to clarify the molecular mechanism by which ZOL directly radiosensitizes tumor cells. Because ZOL-mediated radiosensitization *in vitro* has been reported for various malignancies, including breast cancer, prostate cancer, myeloma, and osteosarcoma [20]−[22], the molecular mechanism described here may also be applicable to these malignancies.

STAT1 is considered to play mutually exclusive roles in tumor cells. Upon activation of interferon (IFN) receptors, STAT1 is phosphorylated at Tyr701 by IFN receptor-associated Janus-activated kinase, and is translocated from the cytoplasm into the nucleus. In the nucleus, STAT1 binds to the cognate transcriptional elements and activates IFN-inducible genes, leading to growth arrest and cell death [23], [24]. On the other hand, STAT1 overexpression is associated with tumor progression [25], [26] and acquisition of RT resistance [19], [25], [27], [28]. Khodarev et al. reported that RT-resistant squamous cell carcinoma (SCC) cells overexpressed STAT1 [27] and suppression of STAT1 recovered RT sensitivity [28]. Similar results were observed in clinically resected RCC tissues and cultured RCC cells [19]. The current study confirmed the role of STAT1 in the RT sensitivity of RCC cells by conducting both knockdown experiment in cells overexpressing STAT1 and forced expression experiment in cells lacking STAT1. Targeting STAT1 may be a promising strategy not only against RCC but also other RT-resistant tumors overexpressing STAT1.

Systemic IFN administration has been one of options for metastatic RCC. The tumor-suppressive role of STAT1 in the IFN pathway and the reported association between the lack of STAT1 and the IFN resistance [29] raise the concern that ZOL may diminish IFN response in RCC patients. Regarding this issue, STAT1 overexpression is associated with IFN resistance in SCC cells [25], [28] and a third-generation bisphosphonate, YM529 which has almost equivalent antitumor effect to ZOL, augmented tumor-suppressive effects of IFN on RCC cells [13]. Thus, ZOL is unlikely to compromise IFN response in RCC cells.

ZOL inhibits farnesyl pyrophosphate synthase within tumor cells [30]. Consequently, ZOL inhibits prenylation of small G proteins such as Ras, which relates to tumor intrinsic radioresistance and has been investigated as a molecular target for radiosensitization [31]. Because Ras mediates both Erk and PI3K/Akt pathways, inhibition of these oncogenic pathways are also possible mechanisms underlying the radiosensitizing effect of ZOL. Although Ras inhibition radiosensitizes various types of cancer cells *in vitro* [32], the role of the Erk and PI3K/Akt pathway in radiosensitization appears to depend on the tissue types and cell lines [17], [33]. In the current study, ZOL activated Erk but exerted radiosensitization in 786-O cells, suggesting that the Ras/Erk pathway is not critically involved in the radiosensitization. Since STAT1 inhibits Erk in hepatocellular carcinoma cells [34], ZOL-mediated downregulation of STAT1 may trigger Erk activation in 786-O cells. Interestingly, ZOL repressed Akt activity mainly by downregulating total Akt level in RCC cell lines with high basal Akt activity. It is unclear, however, whether Akt inhibition is responsible for the radiosensitization by ZOL because, remarkably, ZOL inactivated Akt even in Caki-1 cells that were not radiosensitized by ZOL. As the ZOL-mediated downregulation of Akt is also expected to exert antitumor activity, further investigation is warranted to clarify the underlying mechanisms.

Although the current study reports for the first time that ZOL post-transcriptionally downregulates STAT1 in RCC cells, the specific molecular mechanism by which ZOL downregulates STAT1 remains to be clarified. Further understanding of the underlying mechanisms will contribute to the development of novel strategies targeting STAT1.

Because of rapid accumulation into the bone matrix and slow redistribution, ZOL is advantageous at targeting tumors in the bone. Reportedly, ZOL concentration in bone remained high for at least 10 µmol/kg up to 240 days after dosing, with estimated half-lives of 150 to 200 days *in vivo* [35]. Therefore, 48 h exposure to ZOL at 10 or 20 µM, which efficiently radiosensitized RCC cells *in vitro*, could easily be achieved for bone metastasis lesions in a clinical setting. In contrast, a limitation of ZOL as a potential antitumor agent is its low affinity to non-mineralized tissues. According to pharmacokinetic data, plasma ZOL concentrations decline rapidly from the peak value (911±297 µM) to approximately 1% of the peak 24 h after intravenous infusion of the clinical dose of 4 mg in 15 min [36]. As radiosensitizing effects of ZOL on RCC cells after a short exposure (<24 h) at high concentrations (>100 µM) were not evaluated, we cannot estimate the radiosensitizing effects of ZOL on metastases at organs other than bone in a clinical setting. If such effects are present, brain metastases from RCC, which are often managed with radiotherapy, would be a potential condition in which the radiosensitizing effects of ZOL is expected. A previous study indicated that ZOL is not capable to pass the blood brain barrier in healthy subjects [35]. However, the blood-brain barrier is often disrupted in brain metastatic lesions [37], [38], and thus ZOL is expected to reach the brain lesions.

In conclusion, ZOL directly radiosensitizes RCC cells by potentiating the caspase-3-mediated apoptosis pathway. As its underlying molecular mechanism, we demonstrated for the first time that ZOL post-transcriptionally downregulates STAT1 expression. We confirmed that STAT1 plays a key role in RT sensitivity in RCC cells. Since ZOL reportedly radiosensitizes various tumor cells, STAT1 downregulation may be a common cellular response to ZOL in those overexpressing STAT1. Further clinical and translational studies are warranted to explore the unique antitumor activities of ZOL.
